# Characteristics of cardiac involvement in immune-mediated necrotizing myopathy

**DOI:** 10.3389/fimmu.2023.1094611

**Published:** 2023-02-28

**Authors:** Mengyang Liu, Ying Lin, Lingya Qiao, Juan Chen, Qiang Shi

**Affiliations:** ^1^ Department of Neurology, The First Medical Centre, Chinese PLA General Hospital, Beijing, China; ^2^ School of Medicine, Nankai University, Tianjin, China

**Keywords:** immune-mediated necrotizing myopathy (IMNM), cardiac involvement, anti-SRP antibody, Anti-HMGCR antibody, muscle biopsy

## Abstract

**Objective:**

To investigate the characteristics of cardiac involvement due to Immune-mediated Necrotizing Myopathy (IMNM).

**Methods:**

Patients diagnosed with Immune-mediated Necrotizing Myopathy (IMNM) who attended the Department of Neurology and the Department of Rheumatology and Immunology at the First Medical Center of the PLA General Hospital between February 2011 and June 2022 were collected. Clinicopathological diagnosis of IMNM was performed according to the criteria established by the European Neuromuscular Center (ENMC). All patients underwent muscle biopsy and Myositis-specific antibodies (MSAs) testing. Information included age, gender, disease duration, intramuscular and extramuscular manifestations, laboratory findings (including creatine kinase, lactate dehydrogenase levels, troponin T, myoglobin and atrial natriuretic peptide), electromyography, skeletal muscle pathology and immunohistochemical staining.

**Results:**

A total of 57 patients were included in this study. Of the serological tests, 56.1% (32/57) were positive for SRP, 21.1% (12/57) were positive for HMGCR and 22.8% (13/57) were seronegative. Thirty patients (52.6%, 30/57) presented with varying degrees of cardiac involvement. We performed ECG in 23 patients and found 6 patients with arrhythmia (26.1%), 12 patients with myocardial ischemia (52.2%), and 7 patients with acute coronary syndrome (ST elevation and non-ST elevation myocardial infarction) (30.4%), and 4 patients with left axis deviation or left ventricular high voltage, suggesting left ventricular hypertrophy (17.4%). Cardiac ultrasound was performed in 14 patients and 3 showed pericardial effusion (21.4%); Decreased left ventricular ejection fraction and atrial enlargement were 2 each; 8 showed a decrease in left ventricular diastolic function (57.1%). In addition, one patient had myocardial edema.

**Conclusion:**

Cardiac involvement is not uncommon in IMNM. However, besides clearly statistically significant differences in the disease course, and in the values of troponin T and myoglobin, our data did not show any statistically significant difference in other features of cardiac involvement between patients with different subtypes of IMNM.

## 1 Introduction

Immune-mediated Necrotizing Myopathy (IMNM) is a rare subgroup of idiopathic inflammatory myopathies manifested by severe proximal limb muscle weakness and elevated serum creatine kinase (CK) levels (1, 2). The concept was first introduced by the European Neuromuscular Centre (ENMC) in 2003 and subdivided in 2016 to include anti- 3-hydroxy-3-methylglutaryl coenzyme A reductase (HMGCR) antibody IMNM, anti-signal recognition particle (SRP) antibody IMNM and serum antibody-negative (Namely, the anti-SRP antibody and the anti-HMGCR antibody were negative) IMNM (3, 4).

IMNM is currently considered to be an autoimmune disease with predominantly muscular lesions, as it rarely presents with extra-muscular involvement (5). However, some believe that in addition to symptoms of skeletal muscle involvement such as muscle weakness and myalgia, IMNM can also present with extra-muscular manifestations such as rash, myocarditis, interstitial lung disease and arthritis. In particular, cardiac involvement is the more common extra-muscular manifestation in anti-SRP antibody myopathies (6). Chest pain, palpitations, congestive heart failure, and changes in the electrocardiogram and echocardiogram were found in 2-40% of patients with anti-SRP-positive myopathy. In contrast, cardiac involvement was rarely reported in patients with anti-HMGCR-positive antibody myopathy as well as antibody-negative myopathy (7–9). Therefore, in this study, we aimed to retrospectively characterize cardiac involvement in a large cohort of IMNM patients from China to draw attention to cardiac involvement in IMNM.

## 2 Information and methods

### 2.1 Clinical information

We collected a total of 1,098 patients diagnosed with idiopathic inflammatory myopathy (IIM) patients seen in the Department of Neurology and Rheumatology Department of the First Medical Center of the PLA General Hospital between February 2011 and June 2022, and a total of 57 patients (5.2%) were further diagnosed with Immune-mediated Necrotizing Myopathy (IMNM). All the clinicopathological diagnoses were according to the European Neuromuscular Center (ENMC) IMNM criteria (3), including age, gender, disease duration, intramuscular and extramuscular manifestations, laboratory findings (including creatine kinase, lactate dehydrogenase, troponin T, myoglobin and brain natriuretic peptide), electromyography, skeletal muscle pathology and immunohistochemical staining. The study was approved by our hospital ethics committee and all patients signed an informed consent form.

### 2.2 Myositis antibody test

Serum samples were collected from patients and stored in a refrigerator at -80°C until testing, myositis-specific antibodies (MSAs) and myositis-associated antibodies (MAAs) was detected by Western blot (Western blot Reference results are shown in **Supplementary Figure 1**). Results according to the colour of antigen band: colourless 0: negative; Very weak colouring (+): critical value; Weak staining +: weak positive; Strong staining ++: positive; The intensity of staining was the same as that of the quality control strip.

### 2.3 Skeletal muscle biopsy

After the informed consent was signed, an open skeletal muscle biopsy was performed under local anaesthesia. The muscle samples were frozen and fixed in liquid nitrogen after being pre-cooled with isopentane. The frozen sections were 8μm thick. Routine histological and enzyme histochemical staining were performed, including hematoxylin-eosin (H&E), modified Gomori staining (MGT), reduced coenzyme I (NADH-TR), succinate dehydrogenase (SDH), adenosine triphosphatase (ATPase), acid phosphatase (ACP), oil red O, Sudan black B (SBB) staining Glycogen staining (PAS), non-specific esterase staining (NSE) and so on. In addition, immunohistochemical staining was performed in some muscle samples, including membrane attack complex (MAC) staining and major histocompatibility complex class I (MHC-1) staining.

### 2.4 Statistical analysis

Spss26.0 software (IBM Corp, Armonk, USA) was used to analyze the data. Categorical variables are expressed as percentages and absolute frequencies, and continuous features are represented by mean ± standard deviation or median (quartile range). Chi-square test or Fisher’s exact test were used to compare categorical data; The quantitative variables of two independent samples were compared by Mann Whitney test or Student t test (double-tailed). The quantitative variables of multiple independent samples were compared by ANOVA, and the significance level was p=0.05. If P<0.05, a pairwise comparison was made and the Bonferroni method was used for correction.

## 3 Results

### 3.1 Demographic and clinical information

Demographic and clinical information for all IMNM patients is shown in **Table 1**. 56.1% (32/57) of the serological tests were positive for SRP, 21.1% (12/57) were positive for HMGCR and 22.8% (13/57) were seronegative. In addition, we detected other myositis-specific and myositis-associated antibodies in these patients, including 13 anti-Ro52 antibody positive (10 were anti-SRP common-positive, and 1 was anti-HMGCR common-positive), 2 anti-Ku antibody positive, and 1 anti-RNP, SAE1 and PL-12 antibody positive respective. The mean age of onset was 49.0 years old (range 18-76 years old) in patients who were also accepted into the group, with no statistically significant difference in age between all groups. The ratio of males to females was 1:2.8 (15:42), with females predominating over all subtype groups.

**Table 1:** Demographic and clinical characteristics of different MSA types in IMNM.

	Total (n=57)	Anti-SRP (n=32)	Anti-HMGCR (n=12)	MSAs Negative (n=13)	P-value
**Age**	49.0	49.4	45.7	51.2	0.59
**Female**	42	25/32	8/12	9/13	0.69
**Disease duration(Month)**	15.2	14.3	29.7	4.2	**0.02**
Clinical features					
Muscle myalgia	23	13/32	3/12	7/13	0.33
Muscle weakness	49	27/32	10/12	12/13	0.73
Joint Pain	15	9/32	2/12	4/13	0.67
Renault Phenomenon	6	3/32	1/12	2/13	0.82
Examination results					
Creatine Kinase (U/L)	6661.3	4078.0	4627.4	14983.7	0.052
Lactate Dehydrogenase (U/L)	868.5	915.3	802.2	803.0	0.85
Troponin T (ng/ml)	0.712	0.932	0.229	0.406	**0.005**
Myohemoglobin (ng/ml)	1773.1	1342.4	594.9	3786.3	**0.02**
BNP (pg/ml)	760.7	964.4	535.6	546.3	0.85
Extramuscular activity (EMA)					
**Cardiac Involvement**	30/57	16/32	8/12	6/13	0.53
ECG	23	11	7	5	N/A
Arhythmia	6/23	4/11	2/7	0/5	0.17
Myocardial Ischemia	12/23	5/11	4/7	3/5	0.82
Myocardial Infarction	7/23	3/11	2/7	2/5	0.87
Left ventricular hypertrophy	4/23	2/11	2/7	0/5	0.78
Cardiac ultrasound	14	10	1	3	N/A
Decreased left ventricular diastolic function	8/14	7/10	0/1	1/3	0.33
Decreased left ventricular ejection fraction	2/14	2/10	0/1	0/3	1.00
Pericardial effusion	3/14	1/10	1/1	1/3	0.18
atrial enlargement	2/14	1/10	0/1	1/3	0.51
MRI (Myocardial edema)	1	1	0	0	N/A
** Cutaneous involvement**	22	15/32	4/12	3/13	0.30
** Pulmonary Involvement**	34	24/32	5/12	5/13	**0.03**

BNP, Brain Natriuretic Peptide; ECG, Electrocardiogram.

The bold values represent a P-value of less than 0.05.

The expanded form of "N/A" represents a meaningless P-value.

In terms of clinical symptoms, We performed a detailed physical examination on all of the patients, and the results were shown 40.4% (23/57) had symptoms of myalgia and 86.0% (49/57) had symptoms of muscle weakness, particularly proximal, which tended to develop symmetrically. The median disease duration was 5 months (range 0.25-204 months) and a mean duration was 15.2 months, 70% had a disease course of more than 3 months. Patients in the antibody-negative group had a shorter disease duration compared to those in the antibody-positive group (4.2 vs 29.7 vs 14.3, p=0.02). In addition, 26.3% (15/57) had joint pain and 10.5% (6/57) had Raynaud’s phenomenon. However, the differences in these symptoms were not statistically significant in any of the groups.

Laboratory results showed a median creatine kinase (CK) level of 3417 U/L (range 667.8-26588 U/L, the normal value is 25-200U/L) with a mean of 6068.9 U/L and a mean lactate dehydrogenase (LDH) level of 868.5 U/L (range 293-4825 U/L, the normal value is 109-245U/L). All patients were examined for laboratory indicators related to cardiac involvement, and among the laboratory indicators of cardiac involvement, the median troponin T was 0.478 ng/ml with a mean value of 0.712 ng/ml (range 0.108-2.800 ng/ml, the normal value is under 0.014ng/ml). Troponin-T levels in anti-SRP antibody myopathy were significantly higher than anti-HMGCR antibody myopathy as well as antibody-negative myopathy (0.932 vs 0.229 vs 0.406 ng/ml, p=0.005); the median myoglobin was 1035.0 ng/ml with a mean value of 1773.1 ng/ml (range 100.0-22366 ng/ml, the normal value is under 72ng/ml), the MSAs-negative myopathy had a higher myoglobin level than other two MSAs positive myopathy (1342.4 vs 594.9 vs 3786.3 ng/ml, p=0.02); and the brain natriuretic peptide median was 365.2 pg/ml with a mean of 760.7 pg/ml (range 164.3-3393 pg/ml, the normal value is under 88pg/ml). Electromyography results showed myogenic damage in all patients.

### 3.2 Skeletal muscle pathology

**Figure 1:**
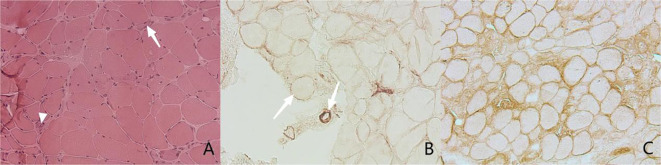
Muscle pathology. **(A)** Hematoxylin and Eosin staining (H&E), original magnifification x200. The image shows necrotic fibers (arrow) and the regenerated muscle fibers (arrow head). **(B, C)** Immnunochemical staining, original magnifification x200. **(B)** shows MAC deposition in sarcolemma of non-necrotic fiber (arrow) and endomysial capillaries (arrow head), While **(C)** indicates diffused expression of MHC class I in sarcolemma of necrotic fiber.

To validate the pathological diagnosis of necrotizing myopathy, muscle biopsies were performed on all patients in our cohort (**Figure 1**), and the muscle specimens were pathologically evaluated. It showed that all the muscle specimens (100%) were characterized by IMNM with varying degrees: H&E staining indicated significant muscle fiber necrosis and muscle fiber regeneration with little or no inflammatory cell infiltration; in addition, We performed immunohistochemical staining of muscle samples from three of these patients, showed positive membrane attack complex (MAC) deposition in non-necrotic muscle fibers and endomysial capillaries in two of them (66.7%), as well as diffused expression of major histocompatibility complex (MHC) class I in non-necrotic muscle fibers in another patient (33.3%).

### 3.3 Clinical features of cardiac involvement

**Figure 2:**
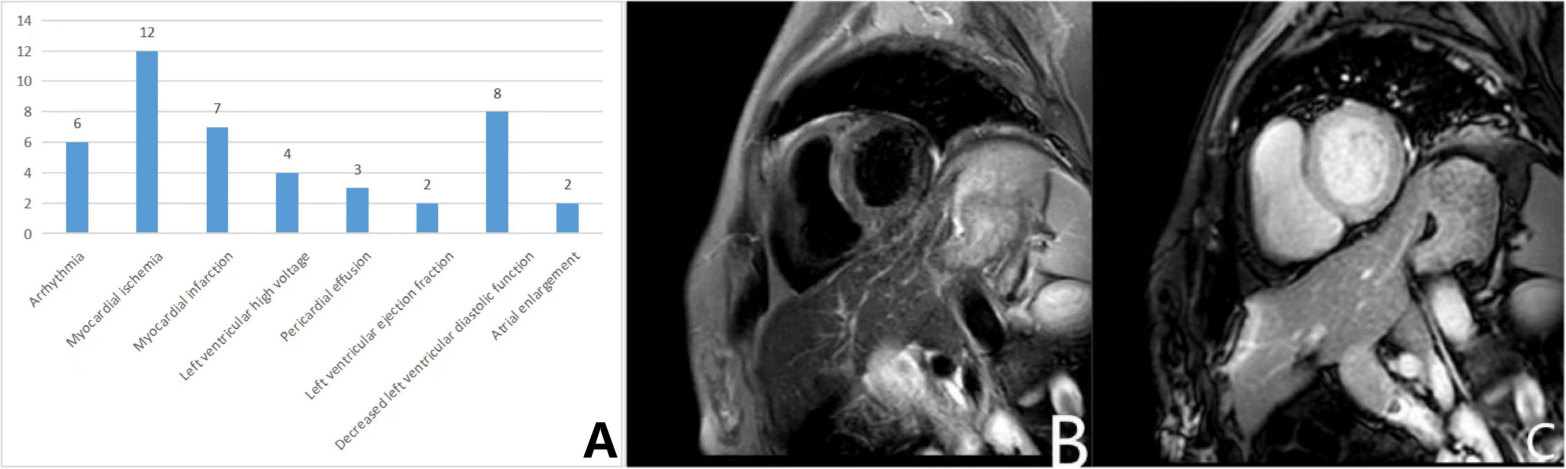
Cardiac involvements in IMNM patients. **(A)** Numbers and types of patients with cardiac symptoms in the IMNM; **(B, C)** Plain and enhanced myocardial MR scan. **(B)** The T2 STIR sequence showed diffused long T2 signal in the left ventricular myocardium; **(C)** No significant myocardial enhancement was seen in the T1 enhancement sequence.

Details of all the IMNM patients with cardiac involvement are listed in the **Supplementary Table**. Statistics showed that a total of 30 patients (52.6%, 30/57) showed varying degrees of cardiac involvement. Among these patients, none reported a cardiac-related medical history. We performed an ECG in 23 patients and found 6 patients with arrhythmia (26.1%), including conduction block, bradycardia, atrial fibrillation, escape, 12 patients with myocardial ischemia (52.2%), and 7 patients with acute coronary syndrome (ST elevation and non-ST elevation myocardial infarction) (30.4%), and 4 patients with left axis deviation or left ventricular high voltage, suggesting left ventricular hypertrophy (17.4%). Cardiac ultrasound was performed in 14 patients and 3 showed pericardial effusion (21.4%); Decreased left ventricular ejection fraction and atrial enlargement were 2 each; 8 showed a decrease in left ventricular diastolic function (57.1%). In addition, one patient had myocardial edema (**Figure 2**). In this study, the incidences of cardiac symptoms between the different antibody subtypes were not significant different.

## 4 Discussion

To date, studies in the literature on IMNM cardiac involvement had been limited to small cohorts with atypical features (5, 9). Researches had shown that the majority of IMNM cardiac involvement was manifested by arrhythmias as well as heart failure (6, 10–13). Therefore, Zhang et al. defined clinically meaningful cardiac involvement as follows (14): Congestive heart failure meeting Framingham’s criteria (15), or conduction abnormalities requiring antiarrhythmic drugs, implanted pacemakers or implantable cardioverter-defibrillators, supraventricular or ventricular arrhythmias; Lilleker et al. added to the criteria for cardiac involvement Lilleker et al. added the criterion of “pericarditis” to the criteria for cardiac involvement (16). Our study was based on the above criteria and integrated the consultation opinions of the Department of Cardiology of the First Medical Center of the PLA General Hospital, according to the electrocardiogram and cardiac ultrasound results, plus the clinical manifestations of patients, finally summarized the specific manifestations of IMNM cardiac involvement including arhythmia, myocardial Ischemia, myocardial Infarction, left ventricular hypertrophy, decreased left ventricular diastolic function, decreased systolic function (decreased left ventricular ejection fraction), pericardial effusion and atrial enlargement.

We reviewed the previous literature on IMNM cardiac involvement and summarized the results in one table (**Table 2**), found that cardiac involvement in IMNM was mostly present in anti-SRP antibody-positive myopathy (10–13). But the prevalence of cardiac involvement in anti-SRP antibody myopathy was still controversial. Our data showed that the prevalence of cardiac involvement in anti-SRP antibody myopathy was 50% (16/32); in a cohort of 13 patients with anti-SRP antibody positive myopathy, Targoff et al. showed arrhythmias and heart failure in four patients (17); in a study by Hengstman et al. over 50% of patients had abnormal ECGs and a further 12 patients had ultrasound, interestingly, more than half of them also had heart failure (8). A study by Suzuki et al. showed that only 2 patients had cardiac involvement in a cohort of 100 patients (7). And another study showed that the prevalence of cardiac involvement in anti-SRP antibody myopathy was relatively low or almost the same compared to the general population (6).

**Table 2:** Results from previous literature studies.

No.	Country	Year	Author	Main Cardiac involvement	Number of patients	Antibody type	Cardiac involvement rate
1	USA	1990	Targoff	Arrhythmias, Cardiac fibrosis and Congestive heart failure	10	Anti-SRP	40%(4/10)
2	Europe	2006	Hengstman	Myocardial infarction and Heart failure	23	Anti-SRP	>50%
3	Japan	2015	Suzuki	Involvement of myocardium	100	Anti-SRP	2%(2/100)
4	USA	2020	Triplett	Left ventricular diastolic and systolic dysfunction	36	Anti-HMGCR	30.6%(11/36)
5	China	2021	Ma	Arrhythmias, Cardiac fibrosis and Heart failure	16	MSAs Negative	68.8%(11/16)

At the same time, cardiac involvement in anti-HMGCR antibody-positive versus seronegative myopathy had been discussed relatively rarely and had mostly focused on case reports. Our study of four previous reports of cardiac involvement in anti-HMGCR antibody myopathy found that all of these patients had heart failure in the form of systolic and diastolic dysfunction, with only one patient presenting with ECG abnormalities and elevated troponin I levels (9, 18–20); a more recent retrospective study found that 11 of 36 anti-HMGCR antibody-positive patients had left ventricular diastolic dysfunction and 6 showed systolic dysfunction. 6 cases showed systolic dysfunction (21). This was in line with our data. As for seronegative myopathy with cardiac involvement, Takumi et al. reported the first patient with antibody-negative IMNM with cardiac involvement in 2021. This patient presented with severe decompensation of diffuse left ventricular function and muscle necrosis and regeneration (22); Tsang et al. reported a case of antibody-negative IMNM in a patient with concurrent fulminant myocarditis leading to cardiogenic shock and cardiac arrest (23); in addition, in a study of a cohort of 117 IMNM patients from China, Ma et al. found that cardiac ultrasound and cardiac MRI (but not ECG) frequently detected seronegative cardiac involvement in IMNM patients. Also in this study, they found that cardiac MRI was more sensitive in detecting cardiac abnormalities in patients with IMNM (24), and such findings will help us in our future research ideas.

Nevertheless, this study still has some limitations. As this was a retrospective study, there may be information bias and selection bias. Also, the relatively high proportion of patients in the anti-SRP antibody-positive group and the relatively low proportion of patients in the anti-HMGCR antibody-positive group, as well as the antibody-negative group, lead to an increased chance in the results of this study and therefore a larger sample size is needed for validation.

## 5 Conclusion

In conclusion, cardiac involvement is not uncommon in IMNM. However, besides clearly statistically significant differences in the disease course, and in the values of troponin T and myoglobin, our data did not show any statistically significant difference in other features of cardiac involvement between patients with different subtypes of IMNM. Therefore, cardiac screening, assessment, and follow-up should be given more consideration in patients with IMNM, and this in turn will contribute to improved IMNM treatment.

## Data availability statement

The original contributions presented in the study are included in the article/**Supplementary Material**. Further inquiries can be directed to the corresponding author.

## Ethics statement

The studies involving human participants were reviewed and approved by Chinese PLA General Hospital Ethical Review Committee (S2017-070-01) and has therefore been performed in accordance with the ethical standards laid down in the 1964 Declaration of Helsinki and its later amendments. The patients/participants provided their written informed consent to participate in this study.

## Author contributions

All authors contributed to the study conception and design. Material preparation was performed by YL, LQ and JC. The first draft of the manuscript was written by ML and all authors commented on previous versions of the manuscript. All authors contributed to the article and approved the submitted version.
