# Corrigendum: Characteristics of cardiac involvement in immune-mediated necrotizing myopathy

**DOI:** 10.3389/fimmu.2023.1233453

**Published:** 2023-08-21

**Authors:** Mengyang Liu, Ying Lin, Lingya Qiao, Juan Chen, Qiang Shi

**Affiliations:** ^1^ Department of Neurology, The First Medical Centre, Chinese PLA General Hospital, Beijing, China; ^2^ School of Medicine, Nankai University, Tianjin, China

**Keywords:** immune-mediated necrotizing myopathy (IMNM), cardiac involvement, anti-SRP antibody, anti-HMGCR antibody, muscle biopsy

In the published article, there was an error in the **Funding** statement. The original funded project “the National Natural Science Foundation of China (No.81771358)” was mistaken as an ongoing project. Currently the former funded project “the National Natural Science Foundation of China (No.81771358)” had been concluded and should not have appeared in the **Funding** section. The correct **Funding** statement appears below.

