# Supplementary figures and images for: Characteristics of cardiac involvement in immune-mediated necrotizing myopathy
Source: Front Immunol. 2023 Feb 28;14:1094611. doi: 10.3389/fimmu.2023.1094611 (PMC10011453; doi:10.3389/fimmu.2023.1094611)

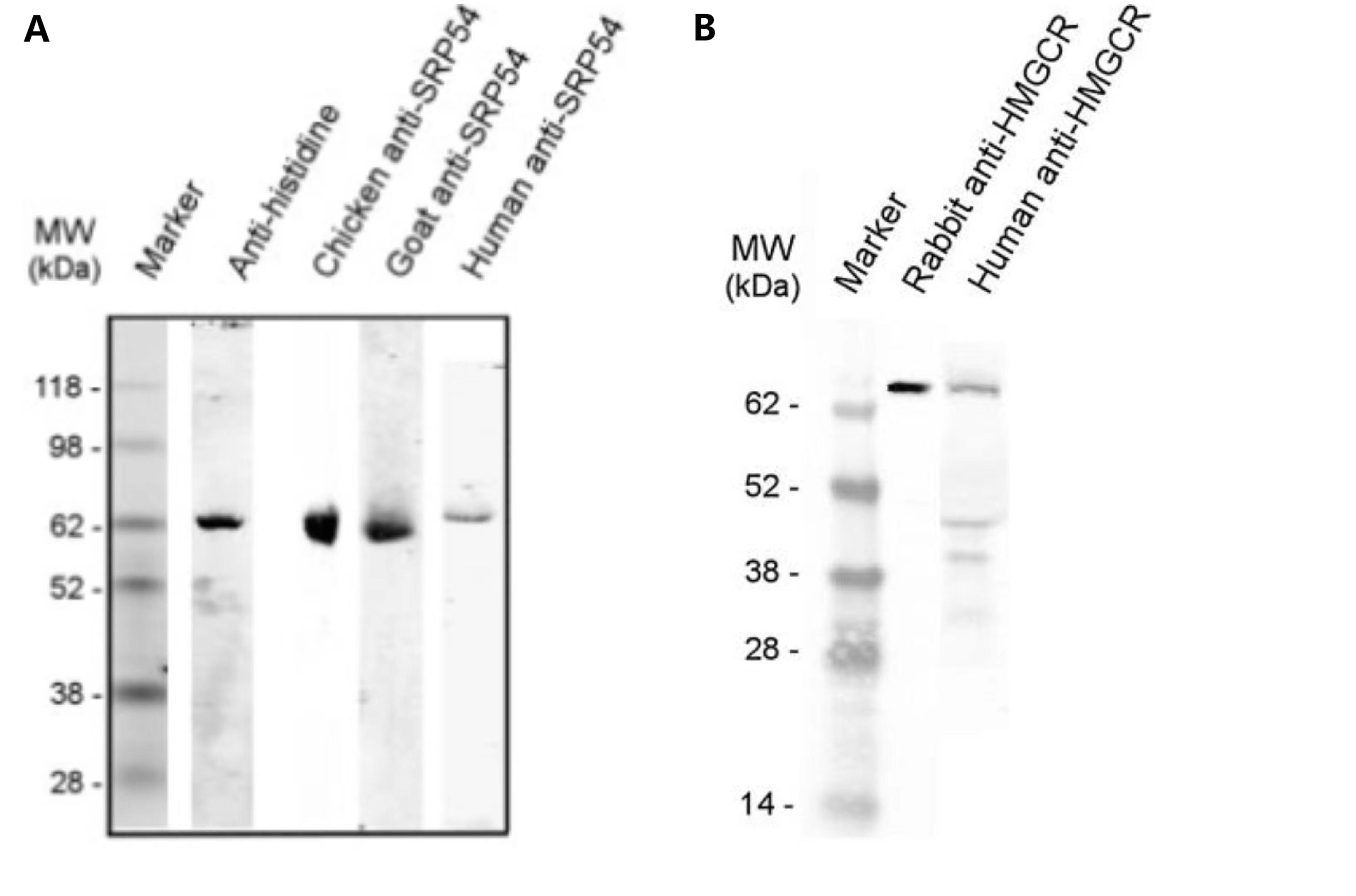

Supplement: Supplementary file 1 [file Image_1.jpeg]
