# Supplementary material for: Characteristics of cardiac involvement in immune-mediated necrotizing myopathy
Source: Front Immunol. 2023 Feb 28;14:1094611. doi: 10.3389/fimmu.2023.1094611 (PMC10011453; doi:10.3389/fimmu.2023.1094611)
Supplement: Supplementary file 2 [file Table_1.docx]

| **Supplementary Table** Characteristics of IMNM patients with cardiac involvements | | | | | | | | | | | |
| --- | --- | --- | --- | --- | --- | --- | --- | --- | --- | --- | --- |
| **No.** | **Sex** | **Age** | **Disease duration(Month)** | **Clinical Features** | **Cadiac Involvement** | **Examination completion** | **MSAs type** | **CK Peak (U/L)** | **Troponin T (ng/ml)** | **Myohemoglobin (ng/ml)** | **BNP (pg/ml)** |
| 1 | M | 44 | 2 | No | Atrioventricular block and Left ventricular hypertrophy | ECG | Anti-HMGCR | 667.8 | N/A | 130.4 | N/A |
| 2 | F | 33 | 20 | Muscle Weakness | Myocardial Ischemia | ECG | Anti-HMGCR | 6667.6 | 0.186 | 633.9 | N/A |
| 3 | F | 54 | 3 | Myalgia and Muscle Weakness | Myocardial Ischemia | ECG | Anti-HMGCR | 6986 | 0.356 | 1059 | N/A |
| 4 | M | 58 | 2 | Muscle Weakness | Myocardial infarction and Left ventricular hypertrophy | ECG | Anti-HMGCR | 5239 | N/A | 185.8 | 1008ng/ml |
| 5 | F | 41 | 60 | Myalgia, Joint pain, Muscle Weakness and Renault Phenomenon | Myocardial Ischemia | ECG | Anti-HMGCR | 8535.8 | 0.197 | 851.2 | N/A |
| 6 | M | 68 | 6 | Muscle Weakness and Joint pain | Bradycardia and Myocardial Ischemia | ECG | Anti-HMGCR and Anti-Ro52 | 3045.8 | 0.180 | 1080 | 225.4pg/ml |
| 7 | F | 25 | 5 | No | Myocardial infarction | ECG | Anti-HMGCR | 3277.3 | N/A | 342.6 | N/A |
| 8 | F | 54 | 5 | Muscle Weakness | Pericardial effusion | Cardiac ultrasound | Anti-HMGCR | 9709 | 0.179 | 1521 | 503.4pg/ml |
| 9 | F | 45 | 2 | Muscle Weakness and Joint pain | Myocardial edema | Myocardial MRI | Anti-SRP | 8023 | 0.315 | 280.4 | 178.8pg/ml |
| 10 | M | 39 | 7 | Muscle Weakness | Myocardial Ischemia and Right bundle branch block | ECG | Anti-SRP | 3052.6 | 0.449 | 1494 | 188.4pg/ml |
| 11 | M | 59 | 5 | Myalgia and Muscle Weakness | Decreased left ventricular diastolic function, Right bundle branch block and extraventricular contraction | ECG and Cardiac ultrasound | Anti-SRP | 2066.1 | 1.110 | 1850 | 1221pg/ml |
| 12 | F | 74 | 12 | Muscle Weakness and Joint pain | Atrial fibrillation, Right atrial enlargement and Myocardial infarction | ECG and Cardiac ultrasound | Anti-SRP | 1840 | 0.130 | 647.6 | N/A |
| 13 | F | 44 | 7 | Muscle Weakness | Decreased left ventricular diastolic function | Cardiac ultrasound | Anti-SRP | 2371 | 0.481 | 1099.96 | N/A |
| 14 | F | 60 | 204 | Muscle Weakness and Joint pain | Myocardial infarction | ECG | Anti-SRP | 10764 | 2.090 | 5614 | N/A |
| 15 | F | 59 | 1 | Myalgia and Muscle Weakness | Decreased left ventricular diastolic function, Pericardial effusion and Myocardial infarction | ECG and Cardiac ultrasound | Anti-SRP | 1756.9 | 0.778 | 1082 | N/A |
| 16 | M | 76 | 2 | Muscle Weakness | Decreased left ventricular diastolic function | Cardiac ultrasound | Anti- SRP and Anti PL-12 | 2676 | N/A | 1754 | 351.6pg/ml |
| 17 | F | 54 | 7 | Myalgia, Joint pain, and Muscle Weakness | Decreased left ventricular diastolic function | Cardiac ultrasound | Anti- SRP and Anti-Ro52 | 4583 | 0.762 | 848.4 | N/A |
| 18 | F | 43 | 36 | No | Escaped rhythm, Atrial fibrillation, Myocardial Ischemia, Valvular regurgitation and Reduced Left ventricular ejection fraction | ECG and Cardiac ultrasound | Anti- SRP and Anti-Ro52 | 1018.4 | 0.184 | 379.8 | 657.8pg/ml |
| 19 | F | 61 | 24 | Myalgia, Joint pain, and Muscle Weakness | Left ventricular hypertrophy | ECG | Anti- SRP and Anti-Ro52 | 1143 | N/A | 553.1 | N/A |
| 20 | F | 70 | 5 | Muscle Weakness | Right bundle branch block , Reduced Left ventricular ejection fraction and Decreased left ventricular diastolic function | ECG and Cardiac ultrasound | Anti-SRP | 2914 | 0.361 | 1141 | N/A |
| 21 | F | 42 | 4 | Muscle Weakness | Myocardial Ischemia | ECG | Anti-SRP | 1668 | N/A | 752.2 | N/A |
| 22 | F | 45 | 6 | Muscle Weakness | Myocardial Ischemia | ECG | Anti-SRP | 6783 | 1.100 | 1060 | N/A |
| 23 | M | 41 | 6 | Myalgia and Muscle Weakness | Decreased left ventricular diastolic function | Cardiac ultrasound | Anti-SRP | 8405 | 1.220 | 1203 | N/A |
| 24 | M | 47 | 12 | Myalgia and Muscle Weakness | Myocardial Ischemia | ECG and Cardiac ultrasound | Anti- SRP and Anti-Ro52 | 3417 | 0.419 | 826.1 | N/A |
| 25 | F | 26 | 4 | Myalgia, Joint pain, Muscle Weakness and Renault Phenomenon | Myocardial Ischemia | ECG | MSAs Negative (Anti-RNP) | 6518 | 0.495 | 1035 | 456.5pg/ml |
| 26 | M | 62 | 1 | Muscle Weakness | Pericardial effusion | Cardiac ultrasound | MSAs Negative | 37952 | 0.208 | 22366 | 365.2pg/ml |
| 27 | M | 38 | 1 | Myalgia and Muscle Weakness | Myocardial infarction | ECG | MSAs Negative | 26588 | N/A | 3340 | N/A |
| 28 | F | 74 | 1 | Myalgia, Muscle Weakness and Renault Phenomenon | Myocardial infarction, Decreased left ventricular diastolic function and Valvular regurgitation | ECG and Cardiac ultrasound | MSAs Negative | 12703 | 0.587 | 1495 | 1324pg/ml |
| 29 | F | 72 | 1 | Muscle Weakness and Joint pain | Myocardial Ischemia and Left atrial enlargement | ECG and Cardiac ultrasound | MSAs Negative (Anti-Ro52) | 6287.8 | 0.543 | 1912 | N/A |
| 30 | F | 66 | 6 | Muscle Weakness and Joint pain | Myocardial Ischemia | ECG | MSAs Negative (Anti-SAE1) | 6811.7 | 0.279 | 2253 | N/A |
| Abbreviations:IMNM Immune-mediated necrotizing myopathy, M Male, F Female, MSAs Myositis specific antibodies, CK Creatine Kinase, BNP Brain Natriuretic Peptide, ECG Electrocardiogram | | | | | | | | | | | |
